# Magnetite nanoparticles facilitate methane production from ethanol via acting as electron acceptors

**DOI:** 10.1038/srep16118

**Published:** 2015-11-12

**Authors:** Zhiman Yang, Xiaoshuang Shi, Chuanshui Wang, Lin Wang, Rongbo Guo

**Affiliations:** 1 Key Laboratory of Biofuels, Qingdao Institute of Bioenergy and Bioprocess Technology, Chinese Academy of Sciences, Qingdao 266101, China

## Abstract

Potential for interspecies hydrogen transfer within paddy soil enrichments obtained via addition of magnetite nanoparticles and ethanol (named as PEM) was investigated. To do this, PEM derived from rice field of Hangzhou (named as PEM-HZ) was employed, because it offered the best methane production performance. Methane production and Fe (III) reduction proceeded in parallel in the presence of magnetite. Inhibition experiments with 2-bromoethane sulfonate (BES) or phosphate showed that interspecies hydrogen transfer and Fe (III) reduction also occurred in methane production from ethanol. 16S rRNA-based Illumina sequencing results showed that *Dechloromonas*, *Thauera*, *Desulfovibrio* and *Clostridium* were the dominant putative Fe (III) -reducers, and that hydrogenotrophic *Methanobacterium* accounted for about 88% of the total archaeal community. These results indicated that magnetite nanoparticles that acted as electron acceptor could facilitate rapid oxidation of ethanol by members of the Fe (III) -reducers in PEM-HZ and establishment of the syntrophic relationship of Fe (III) -reducers with *Methanobacterium* via interspecies hydrogen transfer. Our results could offer a model to understand the microbial interaction with magnetite from a novel angle during methanogenesis.

Ethanol is one of the intermediates from anaerobic degradation of organic substrates. The conversion of ethanol to methane depends on effective interspecies electron transfer (IET) between ethanol oxidizers and methanogens^1^. Two mechanisms for IET have been reported. The former was IET via hydrogen, in which ethanol oxidizers metabolize ethanol and reduce protons to generate H_2_ and the methanogens utilize hydrogen to reduce CO_2_^1^. The latter was direct IET, in which the methanogens can directly use electrons releasing from ethanol oxidizers for reducing of CO_2_ to methane through biological electrical connections^2^.

Magnetite nanoparticles, one of the most common minerals in the soil, are able to facilitate microbial extracellular electron transfer^3^,^4^. This biochemical reaction has strong impact on the conversion of ethanol to methane. Data have revealed that the addition of magnetite to paddy soil had a stimulatory effect on ethanol degradation and methanogenesis, concomitant with the enrichment Fe (III)-reducer *Geobacter* and methanogen *Methanosarcina*^5^. In this case, the researchers speculated that magnetite could act as a conduit of electrons to facilitate direct IET between *Geobacter* and *Methanosarcina*, providing a rapid methane production.

However, besides *Geobacter*, iron oxides added to paddy soil resulted in the enrichment of other Fe (III)-reducing bacteria, such as *Clostridium*^6^,^7^,^8^. Existing evidence has shown that *Clostridium* can utilize acetate to reduce Fe (III) in the supplementary akaganeite slurry, and produce significant amounts of H_2_ which was subsequently consumed^9^. Although a significant abundance of *Clostridia* were also detected in the magnetite-added paddy soil, researchers focused on only direct IET, without much consideration for interspecies hydrogen transfer^5^. H_2_ might be produced and subsequently converted to methane by *Methanosarcina*, which was able to perform hydrogenotrophic methanogenesis^10^. In our preliminary experiment significant H_2_ production and *Clostridia* were detected in paddy soil amended with magnetite and ethanol. Additionally, in syntrophic methanogenesis, ethanol oxidation is thermodynamically unfavorable (ΔG_0_′=+9.6 kJ/mol) and can occur only when a very low H_2_ pressure is kept by hydrogen-scavenging methanogens^11^.

Thus, in the light of this context, we hypothesized that magnetite could facilitate methane production from ethanol in other manners in addition to acting as a conduit of electrons. To test this hypothesis, paddy soil enrichments obtained in the presence of magnetite nanoparticles and ethanol (named as PEM) were firstly established in this work. Using the resulted enrichments, we subsequently determined metabolites, Fe (III) reduction, and microbial community structure. Through inhibitor experiments, we then probed the methanogenic mechanisms.

## Results

### Paddy soil enrichments

To investigate the effects of magnetite on methane production from ethanol, PEM was firstly established. The methanogenic characteristics of paddy soil enrichments from different rice filed (GZ, HZ, and TZ) were evaluated. As shown in Supplementary Figure S1 and Table 1, magnetite added to paddy soil led to a significant increase in the maximum methane production rate and a significant reduction in the lag-phase time compared with paddy soil enrichments obtained in the absence of magnetite nanoparticles (named as PEC). PEM-HZ was selected as inoculum for subsequent semi-continuous enrichment cultivations because PEM-HZ showed the shortest lag-phase time with comparable maximum methane production rate.

**Figure 1:**
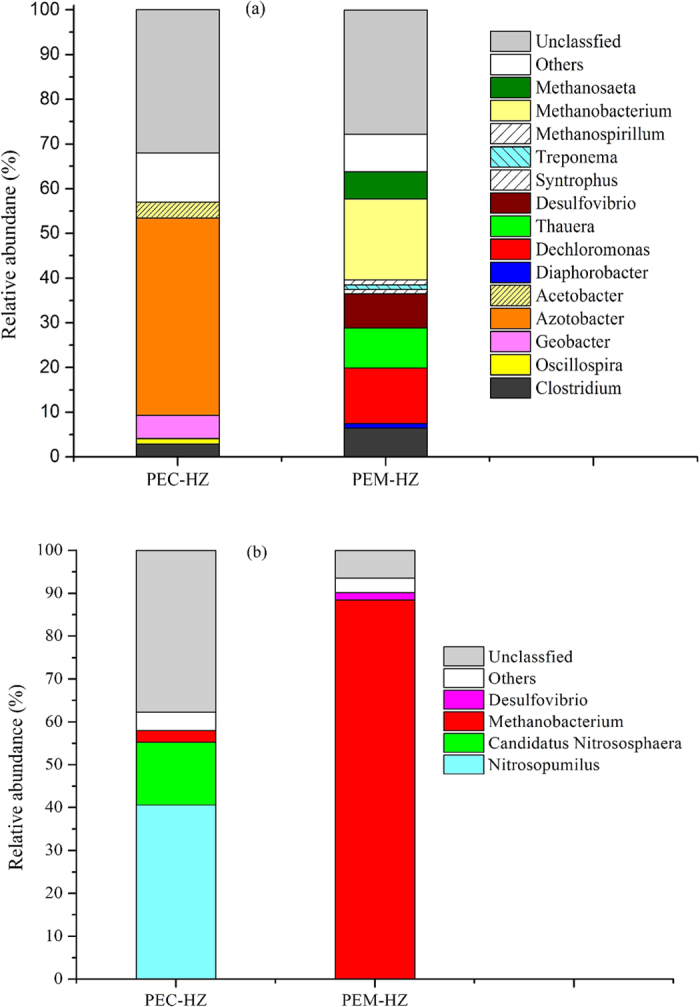
Taxonomic classification of bacteria and communities. The taxonomy of OTU representative sequences was phylogenetically assigned to taxonomic classifications using RDP Classifier and Greengenes with a confidence threshold of 80%. Genus accounting for less than 1% of total composition were classified as “others”. (**a**) Bacteria; and (**b**) Archaea.

Methane production during semi-continuous enrichment cultivation was shown in Supplementary Figure S2 and Table 2. As for each generation of enrichments, the maximum methane production rate of PEM-HZ was higher than that of PEC-HZ. The lag-phase time of methane production in PEM-HZ showed a reduction that was always shorter than that in the corresponding PEC-HZ. However, the methane yield and maximum production rate of PEC-HZ showed a declined tendency with the increase of transfers. Metabolite analysis showed that ethanol were not efficiently removed in PEC-HZ at each generation (Table 3). Interestingly, Table 3 also illustrates that considerable amounts of volatile fatty acids (VFAs) (e.g. butyrate and caproate) were accumulated in PEC-HZ that were higher than those in the corresponding PEM-HZ at each generation. The accumulation of VFAs in PEC-HZ indicated that parts of carbon flux of ethanol might shift towards carboxylic acid production rather than methane production due to the decreased methanogen populations (Fig. 1b). In PEC-HZ,0.07 mmol and 0.01 mmol of H_2_ were produced and subsequently consumed during generation 1 and 2, respectively. It has been reported that anaerobic biotransformation of ethanol can generate acetate, propionate or butyrate^12^, and that consumption of H_2_ and acetate can generate caproate^13^. Thus, it was likely that parts of ethanol was converted to the carboxylic acid in PEC-HZ.

## Structure of microbial communities 

The microbial diversities of the paddy soil enrichments from the second generation were analyzed. A small portion of archaeal and bacterial sequences were recovered, respectively (Fig. 1), due to non-specificity of primer pairs targeting V4 region of bacterial and archaeal 16S rRNA genes. Results from Supplementary Table S2 and Figure S3 showed that magnetite added to the paddy soil altered the structure of microbial communities.

High-throughput Illumina sequencing analysis showed clear differences in communities between PEM-HZ and PEC-HZ. Figure 1a shows the phylogenetic classification of bacterial OTUs at the genus level. 32% (PEC-HZ) and 27.8% (PEM-HZ) of the total reads were not classified at the genus level, indicating that these bacteria are unknown. *Dechloromonas, Thauera, Desulfovibrio* and *Clostridium* in PEM-HZ accounted for 12.4%, 9%, 7.6% and 6.4% of total bacterial sequences, respectively, while those of PEC-HZ were 0.2%, 0.1%, 0.1% and 2.93%. However, a higher percentage of *Azotobacter*, *Geobacter* and *Acetobacter* was observed in PEC-HZ compared with those in PEM-HZ. *Azotobacter* remained as the most abundant (44.2%) in PEC-HZ but decreased to 0.6% in PEM-HZ. Members of *Geobacter* and *Acetobacter* accounted for 5.3% and 3.5% of the bacterial reads sequenced in PEC-HZ, respectively, but the percentage of those in PEM-HZ decreased to 0.7% and 0.03%. *Acetobacter* can transform ethanol to acetate^14^, and *Azotobacter* can utilize acetate to support growth metabolism^15^,^16^. Members of *Azotobacter* genus in PEC-HZ may utilize acetate produced by *Acetobacter* to maintain their growth. Minor genera (relative abundance was less than or equal to 1%) in both two groups were *Oscillospira, Treponema, Syntrophus* and *Diaphorobacter*, but their role in ethanol degradation remained unclear in this work.

Archaeal community analysis showed that *Methanobacterium* accounted for 88.4% in PEM-HZ, while the dominant species for PEC-HZ was *Nitrosopumilus* (40.6%) (Fig. 1b). Only 2.8% of *Methanobacterium* was detected in PEC-HZ. *Nitrosopumilus* has been described as the ammonia-oxidizing archaea^17^, indicating that the ammonia-oxidizing process may occur in PEC-HZ. In addition, 6.1% of acetoclastic methanogen *Methanosaeta* in PEM-HZ was recovered with the bacterial primer set (Fig. 1a).

It should be noted that the maximum methane production rate and methane yield of PEC-HZ significantly declined compared to start-up in the semi-continuous subculture (Table 2). This may be due to the declined *Methanobacterium* populations. It was possible that methanogens were washed out after two generations of subculture. The decreased *Methanobacterium* populations cannot outcompete the dominant species (e.g. *Azotobacter*, *Geobacter*, *Acetobacter* and *Clostridium*) in terms of competition of substrate (e.g. ethanol, acetate and H_2_), causing the suppression of methanogenesis due to competitive exclusion.

## Intermediate metabolites 

**Figure 2:**
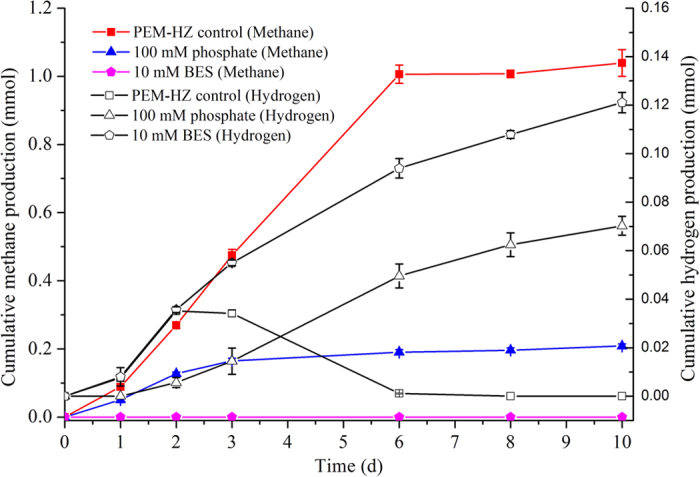
Methane and hydrogen production in the presence of inhibitor. The second generation of PEM-HZ was used as inocula. The inhibitor was added at the beginning of incubation. The addition of BES (10 mM) or phosphate (100 mM) resulted in a significant decrease in methane production and an increase in hydrogen production.

**Figure 3:**
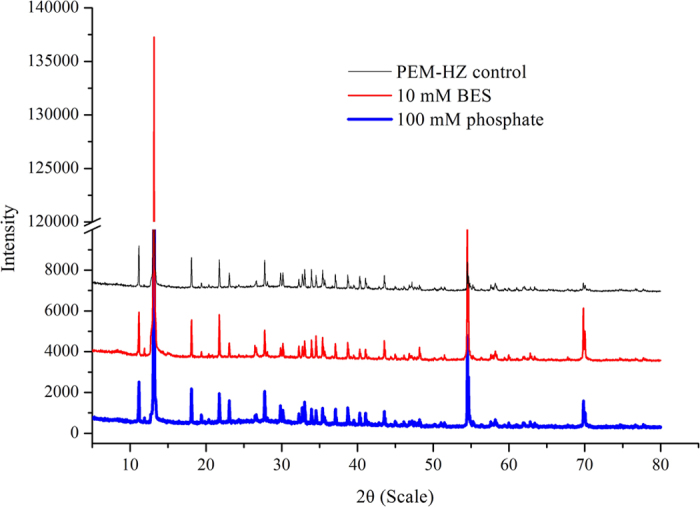
The X ray diffraction spectra of the formed Fe (II)_3_(PO_4_)_2_ at the end of incubation. Magnetite was transformed into Fe (II)_3_(PO_4_)_2_, the pattern of which was consistent with the standard value of Fe (II)_3_(PO_4_)_2_ • 8H_2_O (00-030-0662).

If methane is produced via intermediates, intermediates might be detected and increase with time due to product accumulation after methanogens are inhibited by inhibitors. Thus, both 2-bromoethane sulfonate (BES) and phosphate were used to determine the most probable methanogenic pathways. Figure 2 shows that 0.035 mmol H_2_ was produced and then consumed in PEM-HZ control group (Group I). In group III, BES completely inhibited methane production by PEM-HZ, accompanied with significant amounts of H_2_. Besides small amounts of methane, considerable amounts of hydrogen were produced in group II, indicating that hydrogenotrophic methanogenesis might be partially inhibited by 100 mM phosphate. This was not in agreement with Conrad′s observation^18^, which showed that phosphate can specifically inhibit acetoclastic methanogenesis rather than hydrogenotrophic methanogenesis. Additionally, although Fe (II) concentrations throughout the incubations were below 0.2 mM in three groups (Supplementary Figure S4), X-ray diffraction (XRD) analysis showed that magnetite was transformed into Fe (II)_3_(PO_4_)_2_ at the end of incubation (Fig. 3), indicating that the best parts of Fe (III) in magnetite were reduced to Fe (II).

Besides H_2_, volatile fatty acids (VFAs) (e.g. acetate, propionate and butyrate) were detected (Supplementary Figure S5), while no formate was detected in three groups. On one hand, acetate was accumulated to maximum concentrations of 3.2 mM, and subsequently consumed in group I. In group II and III, acetate was accumulated to maximum concentrations of 2.89 to 6.92 mM at the end of incubation (Supplementary Figure S3). On the other hand, propionate (1–1.52 mM) and n-butyrate (0.5–0.76 mM) accumulated at early stage of incubation in three groups, indicating that methanogenesis and net accumulation of propionate and n-butyrate may proceed in parallel. The produced propionate and n-butyrate were completely depleted at the end of the incubation in three groups. Comparative analysis showed that these acids were further metabolized to acetate in group II and III.

## Discussion

The results showed that magnetite addition to paddy soil altered the microbial ecosystem, accelerated methane production, and led to the dominance of hydrogenotrophic methanogenesis. In this work, magnetite that acted as electron acceptor could facilitate methane production from ethanol by PEM-HZ. This presented an apparent discrepancy with literature reports^5^, which have shown that magnetite that could act as a conduit of electrons facilitated methanogenesis in paddy soil amended with ethanol. This discrepancy might be attributed to the difference in communities between this work and the previous reports. Although *Geobacter* can establish a syntrophic relationship with *Methanosaeta* for converting ethanol to methane via direct IET^2^, only 0.65% of *Geobacter* and 6.1% of *Methanosaeta* were recovered, suggesting that DIET in *Geobacter* might not be the predominant mechanism for methanogenesis in PEM-HZ. Members of *Methanosaeta* genus detected in PEM-HZ might be involved in conversion of acetate to methane as revealed by inhibition experiment with phosphate (Fig. 2 and Supplementary Figure S5).

In the presence of magnetite nanoparticles, Fe (II) formation and methane production were detected (Figs 2 and 3). The Fe (III) reduction was not inhibited by inhibitors (Fig. 3 and Supplementary Figure S4), suggesting that methanogens did not participate in Fe (III) reduction. 16S rRNA-based Illumina sequencing results from PEM-HZ revealed that *Dechloromonas*, *Thauera*, *Desulfovibrio* and *Clostridium* were the most abundant bacterial species. These dominant species have been demonstrated to reduce Fe (III) of iron oxides via respiratory^6^,^7^,^8^,^19^,^20^,^21^. Thus, these species detected in PEM-HZ indicated a strong possibility that they were closely related to reducing Fe (III) of magnetite. It is noteworthy that these Fe (III)-reducers may act as competitors of electron donors (e.g. ethanol and acetate) with *Geobacter*. *Geobacter* cannot outcompete the dominant Fe (III)-reducers in terms of competition of electron donors. Thus, only a small amount of *Geobacter* (0.65%) was enriched due to competitive exclusion in PEM-HZ. Additionally, archaeal community analysis for PEM-HZ revealed that *Methanobacterium* was dominant species, which use H_2_ to reduce CO_2_ and produce methane^22^. These findings showed the possibility that magnetite facilitated the establishment of IET between Fe (III)-reducing bacteria and methanogens based on interspecies hydrogen transfer.

Inhibition experiments showed that the degradation of ethanol occurred, accompanied with the production of H_2_ in the presence of BES or phosphate. This further indicated that methane was likely produced via interspecies hydrogen transfer. It is known that syntrophic oxidation of ethanol becomes thermodynamically favorable reaction only when H_2_ concentration must be maintained very low by hydrogenotrophic methanogens^1^. However, in the presence of magnetite, significant amounts of H_2_ and Fe (II) were formed in group II and III. This suggested that magnetite acted as electron acceptor may facilitated syntrophic ethanol oxidation in a manner similar to Jiang’s report^9^, which observed that considerable amounts of hydrogen gas were produced by acetate oxidizing and Fe (III)-reducing bacteria in the presence of akaganeite and hypothesized that when Fe (III) acted as the electron acceptor, anaerobic acetate-oxidation was an easy oxidation access to acetate with production of H_2_.

Therefore, these observations, combined with the observation that methane production and Fe (III) reduction proceeded in parallel in group I, suggested that, magnetite nanoparticles that acted as electron acceptor could facilitate rapid oxidation of ethanol by members of the Fe (III)-reducing bacteria in PEM-HZ and establishment of the syntrophic relationship of Fe (III)-reducers with *Methanobacterium* via interspecies H_2_ transfer. Similar mechanism might also occur in conversion of the intermediates (e.g. propionate, n-butyrate and caproate) of ethanol degradation to methane. This may result in a rapid methanogenic reaction as observed in this work.

Our results could offer a model to understand the microbial interaction with magnetite from a novel angle during methanogenesis.

## Methods

### Paddy soil sample and preparation of magnetite nanoparticles

Three paddy soil samples were collected from Guangzhou (GZ), Hangzhou (HZ) and Taizhou (TZ), China, respectively. Their characteristics were listed in Table 4. Magnetite nanoparticles (8–10 nm) were synthesized according to Kang′s methods^23^, and identified via X-ray diffraction (XRD) analysis.

## Methane production by batch culture. 

A series of batch tests were performed at 30 ^o^C in 60 mL of anaerobic bottles with a working volume of 20 mL, except for test 3 where 40 mL of working volume in 120 mL bottles was employed. Each test was carried out in triplicate without shaking. The mixture containing inocula and medium was loaded into the bottles. The bottles were subsequently purged with high purity N_2_ for 2 min and sealed with butyl rubber stoppers. The medium (pH 7.0) was composed of 20 mM ethanol, and other components were described previously^24^.

In test 1, three paddy soil samples (GZ, HZ and TZ) were assessed to screen the optimum enrichments in terms of methane production performance. Enrichment cultivation was initiated by inoculated 1.5 g-VS of soil samples into an anaerobic fresh medium. Cultivations were performed in the absence and presence of magnetite (20 mM) respectively. In test 2, the resulted enrichments of HZ (PEC-HZ and PEM-HZ) from test 1 were selected to further conduct semi-continuous subcultures. Specifically, as for the first generation of subculture, 5 mL of PEM-HZ from test 1 (start-up) were added into fresh medium amended with 20 mM magnetite. When methane production reached to the highest yield, the second generation of subculture was initiated by transferring the first generation enrichments (5 mL) to fresh medium supplemented with 20 mM magnetite. The same procedures for two generations of subculture of PEC-HZ were carried out in the absence of magnetite.

In test 3, Methanogenic pathways of PEM-HZ were determined by inhibitor addition (Supplementary Table S1). In inhibition experiments, 0.14 g-VS/L of PEM-HZ from the second generation was grown in the fresh medium containing 20 mM magnetite. The inhibitor was added into the medium at the beginning of incubation. In group II, acetoclastic methanogenesis was detected by inhibition with phosphate (100 mM), an inhibitor of acetoclastic methanogenesis^18^. In group III, hydrogenotrophic methanogenesis was determined by inhibition with BES (10 mM), a specific inhibitor for methanogens^25^.

## Analytical methods. 

The volume of biogas produced was measured using water displacement method. CH_4_ and H_2_ was periodically analyzed using a gas chromatograph^26^. Ethanol, individual VFAs, TS and VS were determined as described previously^24^. Concentrations of Fe (II) extracted with 0.5 N HCl were determined using a ferrozine method^27^. The modified Gompertz equation was used to calculate the maximum methane production rate (Rm) and the lag-phase time (λ)^26^. The methane yield Ps was calculated by dividing cumulative methane production with the total ethanol used. An XRD spectrum was analyzed by a conventional 2θ/θ method using wide angle X-ray diffraction (Bruker D8 Advance).

## DNA extraction, PCR amplification and 16S rDNA sequencing. 

Samples collected from the second generation of paddy soil enrichments were used for total genomic DNA extraction according to a CTAB/SDS method. DNA was purified, quantified and stored at -20°C before PCR amplification. Amplicon libraries were constructed for Illumina sequencing using primer pairs 515f/806r for bacteria^28^ and 519f/ 825r for archaea^29^. PCR, library construction, Illumina sequencing and sequence analysis were performed as described previously^24^. The sequences were deposited to the GeneBank Sequence Read Archive with accession No. SRR1784993.

## Additional Information

**How to cite this article**: Yang, Z. *et al.* Magnetite nanoparticles facilitate methane production from ethanol via acting as electron acceptors. *Sci. Rep.* **5**, 16118; doi: 10.1038/srep16118 (2015).

## Supplementary Material

Supplementary Information

## Figures and Tables

**Table 1:** Kinetic parameters for methane production.

Group	Ps (mol CH_4_/mol EtOH)	Rm (mL/d)	λ(d)	R^2^
PEC-GZ	1.45	1.3	19.9	0.99
PEM-GZ	1.65	2.0	9.3	0.99
PEC-HZ	1.52	1.9	26.3	0.96
PEM-HZ	1.64	4.5	8.1	0.99
PEC-TZ	1.39	2.1	29.2	0.99
PEM-TZ	1.59	4.5	9.2	0.99

**Table 2:** Methane yield, methane production rate and lag-phase time in the semi-continuous subculture.

	Ps (mol CH_4_/mol ethanol)	Rm (mmol/d)	λ(d)	R^2^
PEC-HZ				
Start-up	1.52	0.08	26.3	0.96
Generation l	0.29	0.004	4.9	0.95
Generation 2	0.38	0.01	8.9	0.99
PEM-HZ				
Start-up	1.64	0.20	8.1	0.99
Generation l	1.52	0.11	3.0	0.99
Generation 2	1.59	0.07	0.9	0.99

**Table 3:** Metabolite analysis in the semi-continuous subculture.

	Ethanol (mM)	Acetate (mM)	Propionate (mM)	Butyrate (mM)	Caproate (mM)
PEC-HZ					
Start-up	0.64 ± 0.10	1.26 ± 0.08	0.26 ± 0.02	0.11 ± 0.08	0.43 ± 0.18
Generation l	8.91 ± 4.64	5.28 ± 0.44	2.07 ± 0.23	1.48 ± 0.09	1.10 ± 0.08
Generation 2	7.55 ± 0.07	1.31 ± 0.10	0.02 ± 0.00	8.30 ± 0.30	4.72 ± 0.00
PEM-HZ					
Start-up	0.59 ± 0.34	0.72 ± 0.29	0.37 ± 0.16	0.15 ± 0.10	0.37 ± 0.27
Generation l	0	5.84 ± 2.55	2.46 ± 1.76	0.54 ± 0.51	0.80 ± 0.33
Generation 2	0	5.06 ± 0.36	0.65 ± 0.54	0.21 ± 0.05	0

**Table 4:** The characteristic of paddy soil sample.

Inoculum source	Total solid (TS) (w/w)	Volatile solid (VS) (w/w)
Guangzhou (GZ)	61.0 ± 0.13%	8.12 ± 0.21%
Hangzhou (HZ)	62.9 ± 0.67%	6.07 ± 0.07%
Taizhou (TZ)	62.32 ± 0.98%	4.34 ± 0.21%
