# Supplementary material for: Magnetite nanoparticles facilitate methane production from ethanol via acting as electron acceptors
Source: Sci Rep. 2015 Nov 12;5:16118. doi: 10.1038/srep16118 (PMC4642290; doi:10.1038/srep16118)
Supplement: Supplementary Information [file srep16118-s1.pdf]

Magnetite nanoparticles facilitate methane production from ethanol via acting as electron acceptors

Zhiman Yang, Xiaoshuang Shi, Chuanshui Wang, Lin Wang, Rongbo Guo*
Key Laboratory of Biofuels, Qingdao Institute of Bioenergy and Bioprocess Technology, Chinese Academy of Sciences, Qingdao 266101, China.
* Corresponding author: guorb@qibebt.ac.cn, Tel. & fax: +86 532 80662708

Supplementary information

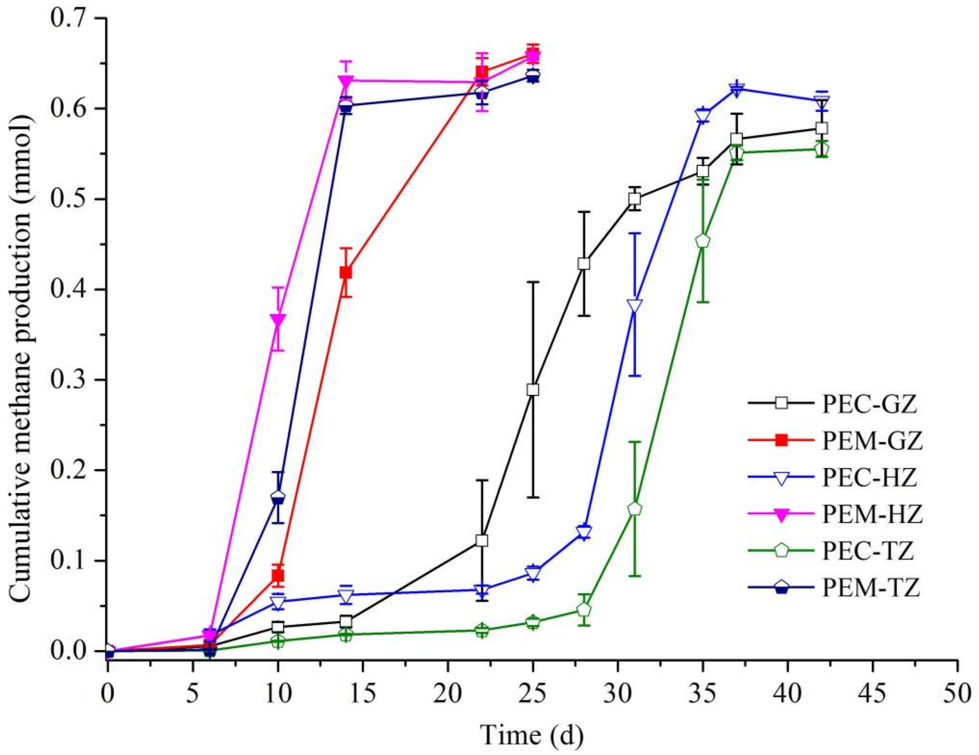

Supplementary Figure S1. Methane production in the batch cultivation using different paddy soils as inocula. Three paddy soil samples were used to select the best enrichments degrading ethanol in the presence of magnetite nanoparticles. CH_4 periodically analyzed by GC. PEM-HZ showed the best methane production performance in terms of methane production rate and lag-phase time.

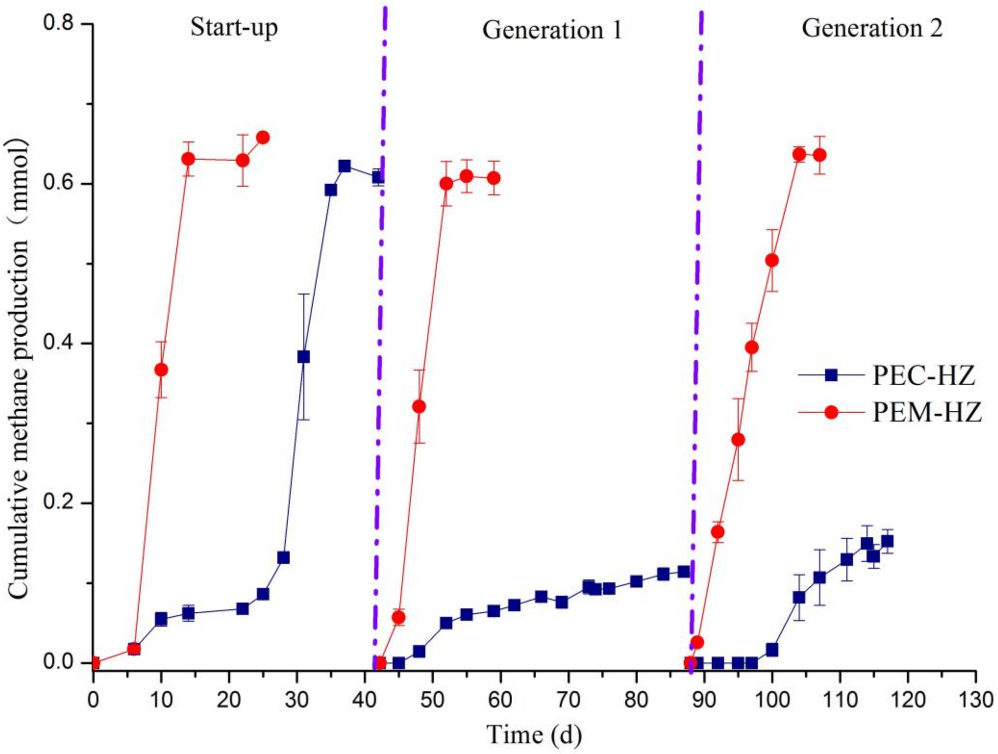

Supplementary Figure S2. Profiles of methane production in the semi-continuous enrichment cultivation. At the start-up stage, 1.5 g-VS of paddy soil (HZ) was amended to 20 mL of sterile medium containing 20 mM of magnetite. When the methane production reached to a plateau, subculture was initiated. 5 mL of the paddy soil enrichments were added in fresh medium same to that used for start-up. Two generations of transfers were conducted. As for the PEC-HZ, the same procedures were performed without magnetite addition. CH_4 was periodically analyzed by GC.

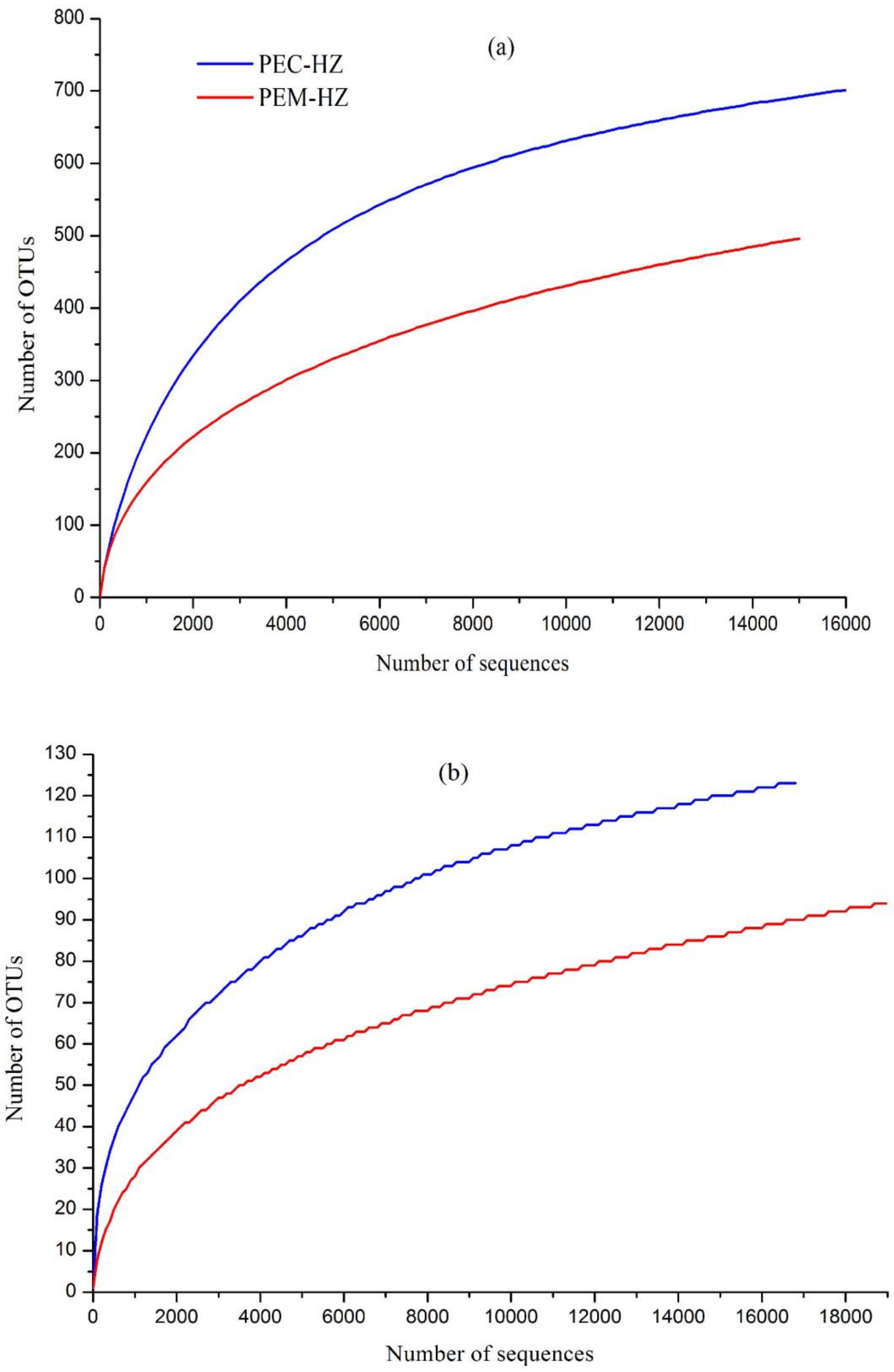

Supplementary Figure S3. Rarefaction curves for both bacteria and archaea communities. The paddy soil enrichments (HZ) obtained from the second generation of subculture were used to analyze the microbial community. The OTUs were defined by 0.03 distance irrespective of the non-specific amplification sequences. (a): Bacteria; and (b) Archaea

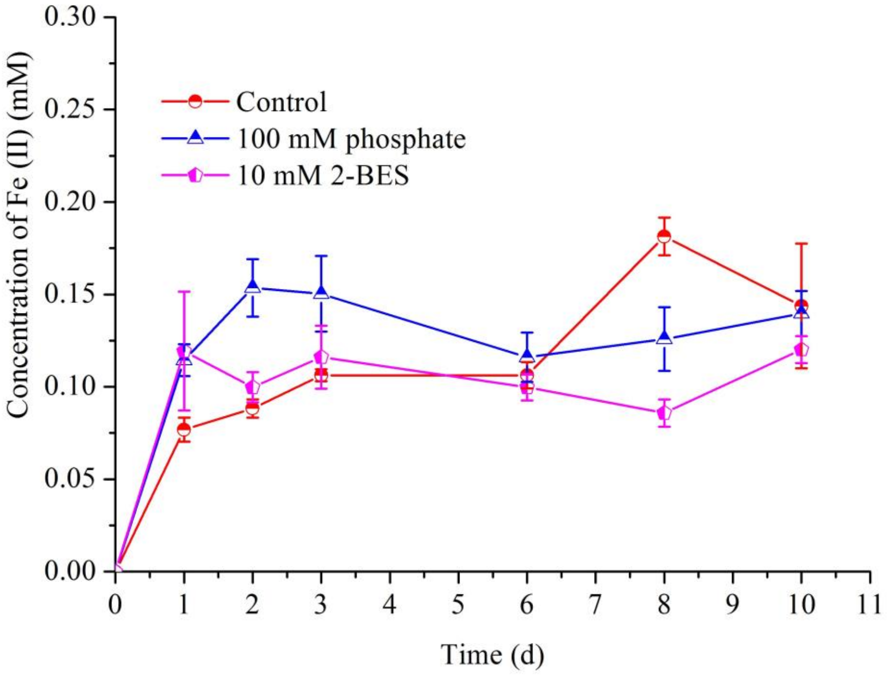

Supplementary Figure S4. Time course of Fe^{2+} concentration in the presence of inhibitors. The concentrations of Fe^{2+} were measured using a ferrozine method. The Fe^{2+} concentrations throughout the incubations were below 0.2 mM in all experimental groups.

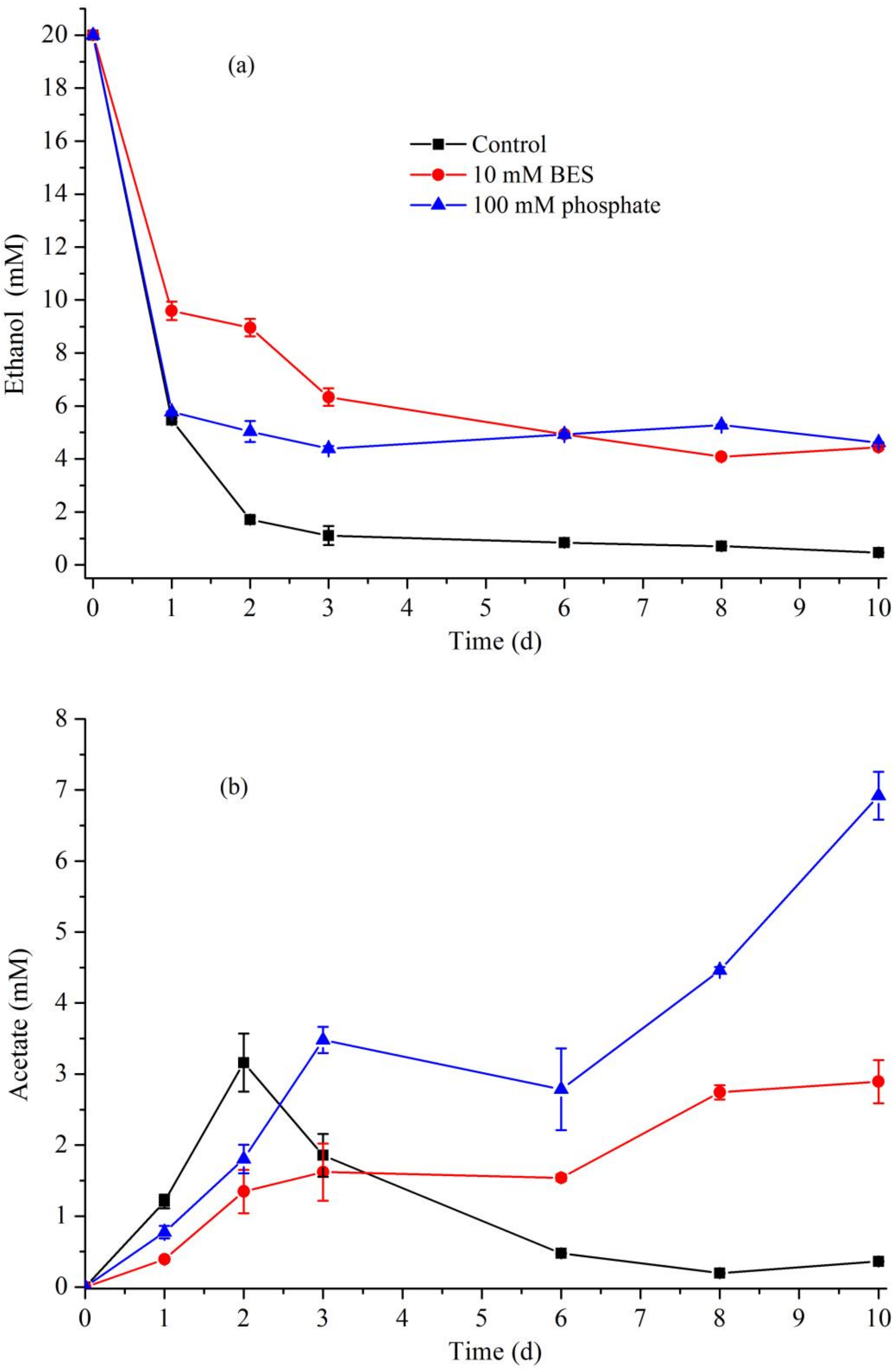

(a)
Control
10 mM BES
100 mM phosphate
Ethanol (mM)
Time (d)
(b)
Acetate (mM)
Time (d)

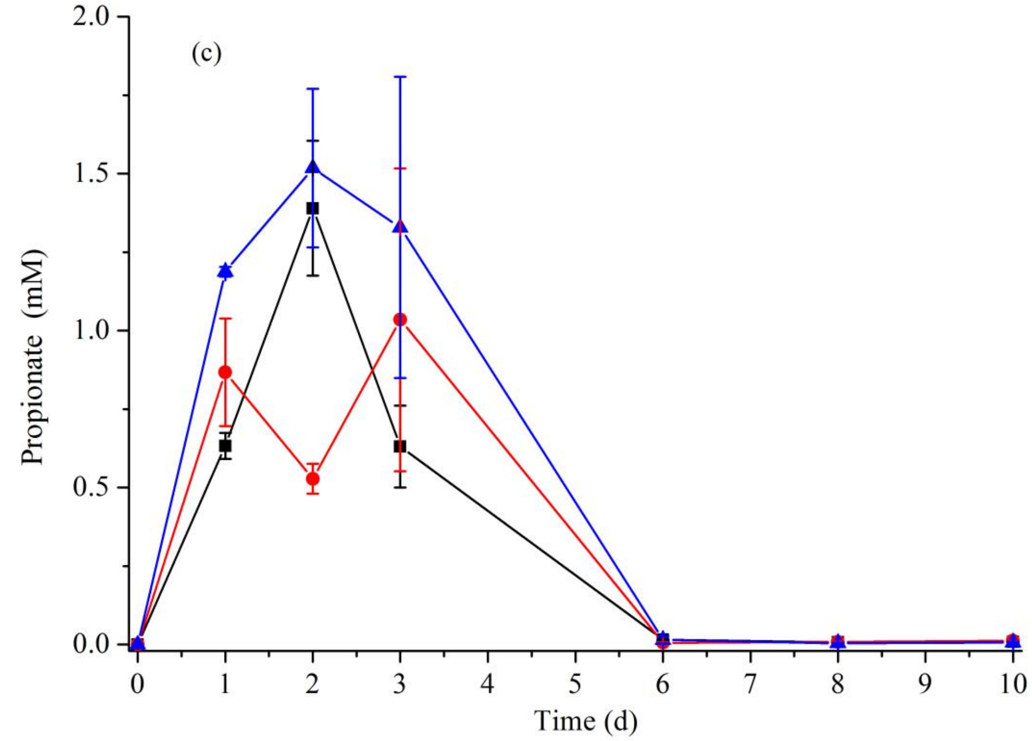

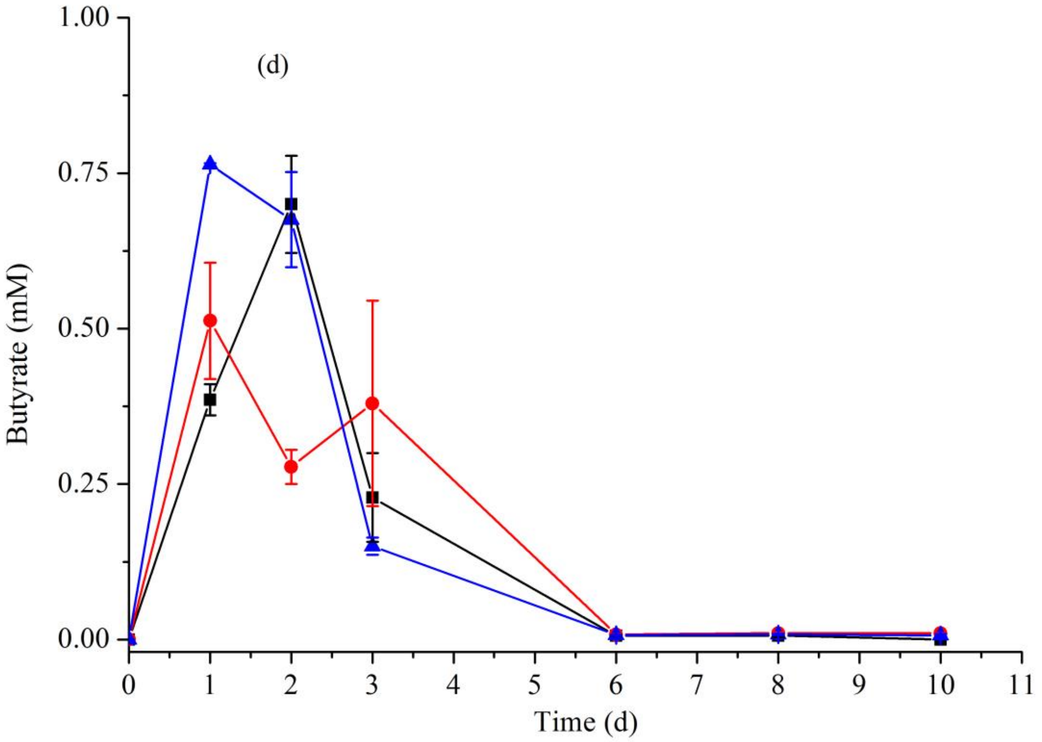

Supplementary Figure S5. Changes of ethanol and volatile fatty acids in the inhibitor-added cultures and the unamended control. The following concentrations were determined: (a) Ethanol; (b) Acetate; (c) Propionate and (d) n-Butyrate. Propionate and n-butyrate were produced and then were degraded in all experimental groups. The addition of BES or phosphate resulted in significant acetate accumulation at the end of incubation.

Supplementary tables

Supplementary Table S1. Description of inhibition experimental groups.

Group	Treatment
I	PEM-HZ control
II	100 mM phosphate
III	10 mM BES

Supplementary Table S2. Comparison of the richness and diversity of 16S rRNA gene libraries based on 0.03 distance irrespective of the non-specific amplification sequences.

	OTUs	Coverage (%)	CHAO1 richness estimation	Shannon diversity
Bacteria				
PEC-HZ	725	96.2	829	3.53
PEM-HZ	496	96.7	661	3.85
Archaea				
PEC-HZ	123	99.3	152	2.25
PEM-HZ	95	99.5	152	0.95
